# Analysis of efficacy and safety for the combination of regorafenib and PD-1 inhibitor in advanced hepatocellular carcinoma: A real-world clinical study

**DOI:** 10.1016/j.iliver.2024.100092

**Published:** 2024-03-27

**Authors:** Zhongchao Li, Jingtao Zhong, Chengsheng Zhang, Bo Zhang, Xuetao Shi, Lei Li

**Affiliations:** Department of Hepatobiliary Surgery, Shandong Cancer Hospital Affiliated to Shandong First Medical University, Jinan 250000, China

**Keywords:** Regorafenib, PD-1 inhibitor, Hepatocellular carcinoma, Combination therapy

## Abstract

**Background and aims:**

Hepatocellular carcinoma (HCC) is a prevalent and deadly disease with limited treatment options. Regorafenib, a tyrosine kinase inhibitor, has shown promise in HCC treatment but faces limitations as a monotherapy. Combining regorafenib with PD-1 inhibitor may improve efficacy and survival outcomes for patients. This retrospective analysis was conducted to explore its efficacy and safety, providing reference experience for better application of this combination therapy.

**Methods:**

This retrospective single-center study evaluated the efficacy and safety of combining regorafenib with PD-1 blockade for patients with HCC. Efficacy was evaluated according to the RECIST 1.1 evaluation criteria. Safety was assessed using CTCAE 4.0. Data was analyzed to compare survival status in different subgroups.

**Results:**

Generally, there were 76 patients with HCC elected to receive the regorafenib plus PD-1 blockade treatment during the study period. The objective response rate was 21.1% (*n* = 16), and the disease control rate was 56.6% (*n* = 43). Median progression-free survival (PFS) was 6.8 months, and median overall survival had not yet been reached. All patients suffered of at least 1 adverse event. Grade ≥3 adverse events occurred in 31.6% of patients (*n* = 24), with the most common being hand-foot syndrome, decreased appetite, and abdominal distension. Subgroup analyses showed no significant differences in PFS based on cirrhosis status or previous treatment lines.

**Conclusion:**

With manageable safety, regorafenib combined PD-1 inhibitor could bring survival benefits for advanced HCC who have received systemic treatment. Further, the Cox analysis showed that HBV infection, metastasis, etc. did not have significant effects on the survival benefits.

## 1 Introduction

Primary liver cancer ranks as the sixth most prevalent cancer worldwide and is the third leading cause of cancer-related death [1]. Hepatocellular carcinoma (HCC) accounts for approximately 75% of primary liver cancer. Unfortunately, most HCC cases are not diagnosed until the middle or late stages, which means that the opportunity for curative surgery has been lost [2,3].

Because of the advancements in targeted therapies and immunotherapy, there has been a continuous increase in systemic treatment options for HCC in recent years. Currently, sorafenib, lenvatinib, and atezolizumab combined with bevacizumab are the first-line drugs approved for advanced HCC. In addition, various tyrosine kinase inhibitors (TKIs) and immune checkpoint inhibitors (ICIs) have shown promising potential in second-line treatment [4]. The combined treatment regimen based on targeted agents and ICIs is the main trend of current research. However, despite these developments, the survival benefits for advanced HCC patients remain severe. The exploration of optimal second-line treatment strategies for patients who have previously received systemic therapy falls far behind the first-line setting, underscoring the urgent need to continue exploring new treatment strategies that can provide better survival outcomes for these individuals.

Regorafenib, a multi-targeted tyrosine kinase inhibitor, inhibits tumor angiogenesis and hinders the metastasis and diffusion of tumor cells. It has been approved for the treatment of metastatic colorectal cancer and advanced gastrointestinal stromal tumors [5]. In addition, the results of the global phase 3 clinical study RESORCE trial showed that compared with a placebo, regorafenib can significantly prolong the survival of patients after the occurrence of tumor progression following sorafenib treatment [6]. Although regorafenib monotherapy can effectively improve the survival of patients, it still faces issues such as low effectiveness and short-term disease progression. Theoretical basis and clinical evidence both indicate that use of a TKI plus ICI can demonstrate synergistic effects, further improving survival benefits in some patients [7].

Therefore, regorafenib combined with a PD-1 inhibitor may further improve efficacy and provide survival benefits to patients. On the basis of these issues, we conducted a retrospective analysis of the treatment regimen of regorafenib plus a PD-1 inhibitor at our center to explore the efficacy and safety in a real-world setting, providing a reference experience for better application of this combination therapy.

## 2 Methods

### 2.1 Study design and participants

This is a single-center retrospective study conducted to evaluate the efficacy and safety of regorafenib + PD-1 inhibitor for advanced HCC. The study includes patients who meet all the following criteria: age ≥18 years; with a radiographic or pathologic diagnosis of HCC; Child-Pugh class A or B; at least one measurable lesion according to RECIST 1.1 criteria; treatment experience of at least one systemic therapy. All patients participated in the study voluntarily and signed an informed consent form. Exclusion criteria: patients with malignancies of the bile ducts, patients with poor physical condition, and patients who passed away shortly after treatment. The detailed study criteria are available in the Supplementary data.

### 2.2 The therapy regimen and evaluation of efficacy and safety

All patients received at least one cycle of systemic treatment with regorafenib + PD-1 inhibitor as the main treatment. The names and doses of the PD-1 inhibitors are shown in Tables S1 and S2. The efficacy evaluation information includes progression-free survival (PFS), overall survival (OS), objective response rate (ORR), and disease control rate (DCR). Efficacy evaluation was performed using the RECIST 1.1 evaluation criteria. Safety evaluation includes the incidence of grade 1–2 adverse events and the incidence of grade 3–4 adverse events. Safety assessment was performed using the Common Terminology Criteria for Adverse Events (version 4.0).

### 2.3 Multivariate analysis of characteristics and therapeutic response predictions

Baseline characteristics and disease progression-related data of patients were collected under the requirements of the clinical study. The study conducted stratified comparisons of PFS and OS based on factors such as the patient's hepatitis status, presence of metastases, number of prior systemic treatments, and whether they underwent combination local treatment. The study adhered to the standards including the use of standardized evaluation criteria for efficacy and safety assessment.

### 2.4 Statistical analysis

The sample size was calculated by the performance goal method. The performance goal of a 63% rate of non-progress was according to the recent trial by Mao et al., and the non-inferiority margin was set at 20% [8]. In addition, we consider that 5% of the subjects dropped out during the study. Ultimately, a minimum target sample size of 51 was established. The baseline information of the patients was analysed using statistical descriptive methods and presented in the form of mean and median values. The median PFS and OS of the patients were calculated using the Kaplan-Meier (K-M) method, and the survival status of patients in different subgroups was compared using the log-rank test. All statistical analyses were performed using SPSS (IBM) and R, and all *p*-values of <0.05 by two-tailed test were considered significant.

## 3 Results

### 3.1 The study process and inclusion characteristics

**Fig. 1:**
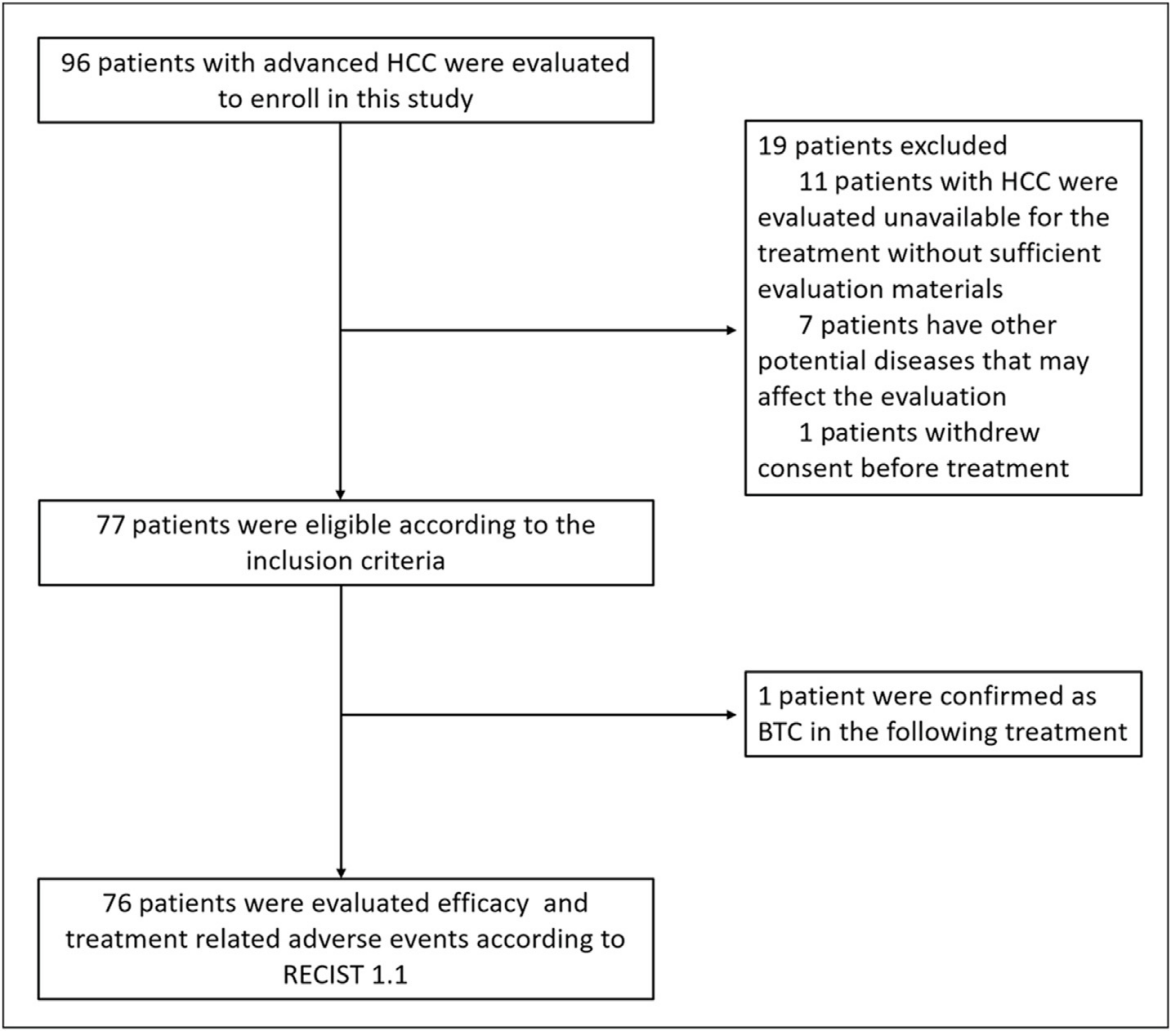
The follow chart of the study.

Between December 1, 2018, and August 1, 2020, a total of 96 patients were enrolled in the study. Of those, 19 patients were excluded because of insufficient efficacy assessment data, potential disease-related factors that could impact treatment, or withdrawal from the study. The detailed treatment information for 76 patients was ultimately evaluated in the study. The selection chart is displayed in Fig. 1.

Within the patient population ultimately included in the study, 59 patients (77.6%) had chronic hepatitis B, and 27 patients (35.5%) displayed signs of cirrhosis. Sixty-nine patients (90.8%) were at ECOG-PS 1, while based on Child-Pugh grading, 45 patients (59.2%) were classified as Child-Pugh A. Twenty-six patients (34.2%) had extrahepatic metastases. According to the inclusion criteria of the study, 61 patients (80.3%) had previously received one cycle of systemic treatment, while 12 patients (15.8%) had received two or more types of systemic treatment. Regarding previous treatment regimens, 60 patients (78.9%) had been treated with sorafenib, while the rest had received lenvatinib (*n* = 6, 7.9%), apatinib (*n* = 4, 5.3%), or other types of treatment (*n* = 1, 1.3%). Twenty-nine patients (38.2%) received local treatment simultaneously during their treatment, and 15.8% of patients received local treatment previously. The detailed information on the enrolled patients is shown in Table 1.

**Table 1:** Baseline characteristics of patients before the combination immunotherapy.

	All (*n* = 76)
Age (median, range) (yr)	54.66 ± 9.60
Sex	
male, *n* (%)	68 (89.5)
female, *n* (%)	8 (10.5)
BMI (mean ± SD)	23.61 ± 3.23
Hepatitis (HBV) infection, *n* (%)	59 (77.6)
Liver cirrhosis, *n* (%)	27 (35.5)
Child-Pugh	
A	45 (59.2)
B	31 (40.8)
ECOG performance, *n* (%)	
0	2 (2.6)
1	69 (90.8)
2	5 (6.6)
BCLC stage (%)	
2	7 (9.2)
3	15 (19.7)
4	53 (69.7)
Extrahepatic metastasis, *n* (%)	26 (34.2)
Number of previous treatment regimens, *n* (%)	
0	3 (3.9)
1	61 (80.3)
≥2	12 (15.8)
Previous surgical treatment, *n* (%)	22 (28.9)
Previous treatment regimens	
Sorafenib, *n* (%)	60 (78.9)
Lenvatinib, *n* (%)	6 (7.9)
Apatinib, *n* (%)	4 (5.3)
Others, *n* (%)	1 (1.3)
Combined with local treatment, *n* (%)	29 (38.2)

Abbreviation: BMI, body mass index; ECOG, Eastern Cooperative Oncology Group; HBV, hepatitis type B virus; BCLC: Barcelona Clinic Liver Cancer.

### 3.2 Patient medication status, efficacy and safety assessment

**Fig. 2:**
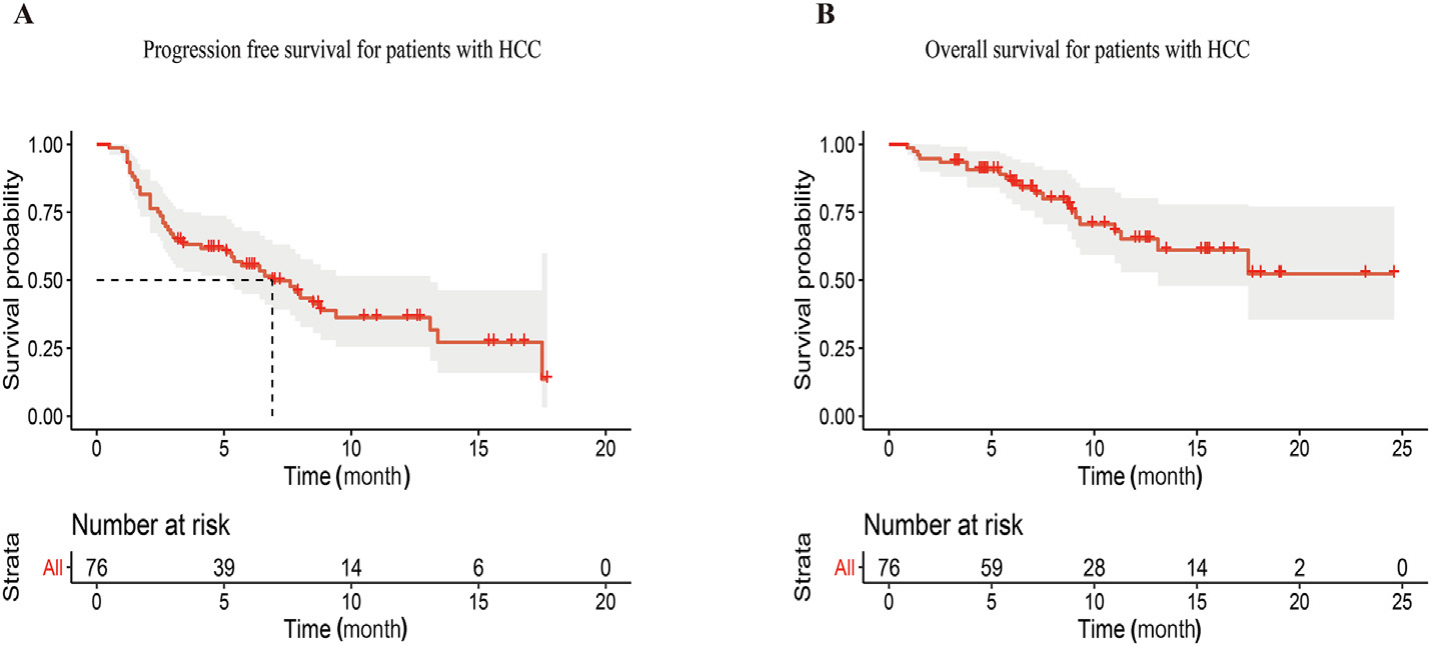
Progressed free survival and overall survival of the patients treated with regorafenib combined with PD-1 inhibitors. (A) The progressed free survival of enrolled patients. (B) The overall survival of enrolled patients.

According to RECIST 1.1 evaluation criteria, one patient achieved complete remission, 15 patients achieved partial remission, 27 patients had stable disease, and 33 patients experienced disease progression. Overall, the objective response rate was 21.1% (*n* = 16), and the disease control rate was 56.6% (*n* = 43). The clinical efficacy is displayed in Table 2. As of the follow-up time, the median PFS was 6.8 months, while the median OS has not been reached yet (Fig. 2). The median follow-up time was 11.0 months.

**Table 2:** Clinical efficacy of regorafenib plus PD-1 inhibitor for HCC patients.

Investigator review according to RECIST 1.1	All (*n* = 76)
Objective response rate, *n* (%)	16 (21.1)
Complete response, *n* (%)	1 (1.3)
Partial response, *n* (%)	15 (19.7)
Stable disease, *n* (%)	27 (35.5)
Progressive disease, *n* (%)	33 (43.4)
Disease control rate, *n* (%)	43 (56.6)
Progression-free survival (months)	6.8 ± 1.19
Overall survival (months)	–
Median follow-up time (months)	11.0 ± 1.82

Abbreviation: RECIST 1.1, response evaluation criteria in solid tumors, version 1.1.

Among the treated patients, 38 patients required drug adjustment because of adverse events. All patients experienced varying degrees of adverse events, with 52 patients experiencing grade 1–2 adverse reactions and 24 patients experiencing grade 3–4 adverse events. The most common grade 1–2 adverse events were hand-foot syndrome, decreased appetite, and abdominal distension, while the most common grade 3–4 severe adverse events were decreased appetite, hand-foot syndrome, and hypoalbuminemia (Table 3).

**Table 3:** Treatment related adverse events in patients treated with Regorafenib combined with PD-1 antibody.

	All treated patients (*n* = 76)	
	Grade 1/2, *n* (%)	Grade 3/4, *n* (%)
Palmar–plantar erythrodysesthesia syndrome	15	3
Decreased appetite	12	5
Abdominal distention	11	2
Vomiting	9	2
Abdominal Pain	8	0
Decreased platelet count	6	1
Diarrhea	5	0
Hypertension	4	0
Hypoalbuminemia	4	3
Cough	3	0
Digestive tract hemorrhage	2	1
Increased blood bilirubin	2	1
Rash	2	0

### 3.3 Subgroup analyses and therapeutic response predictions

**Fig. 3:**
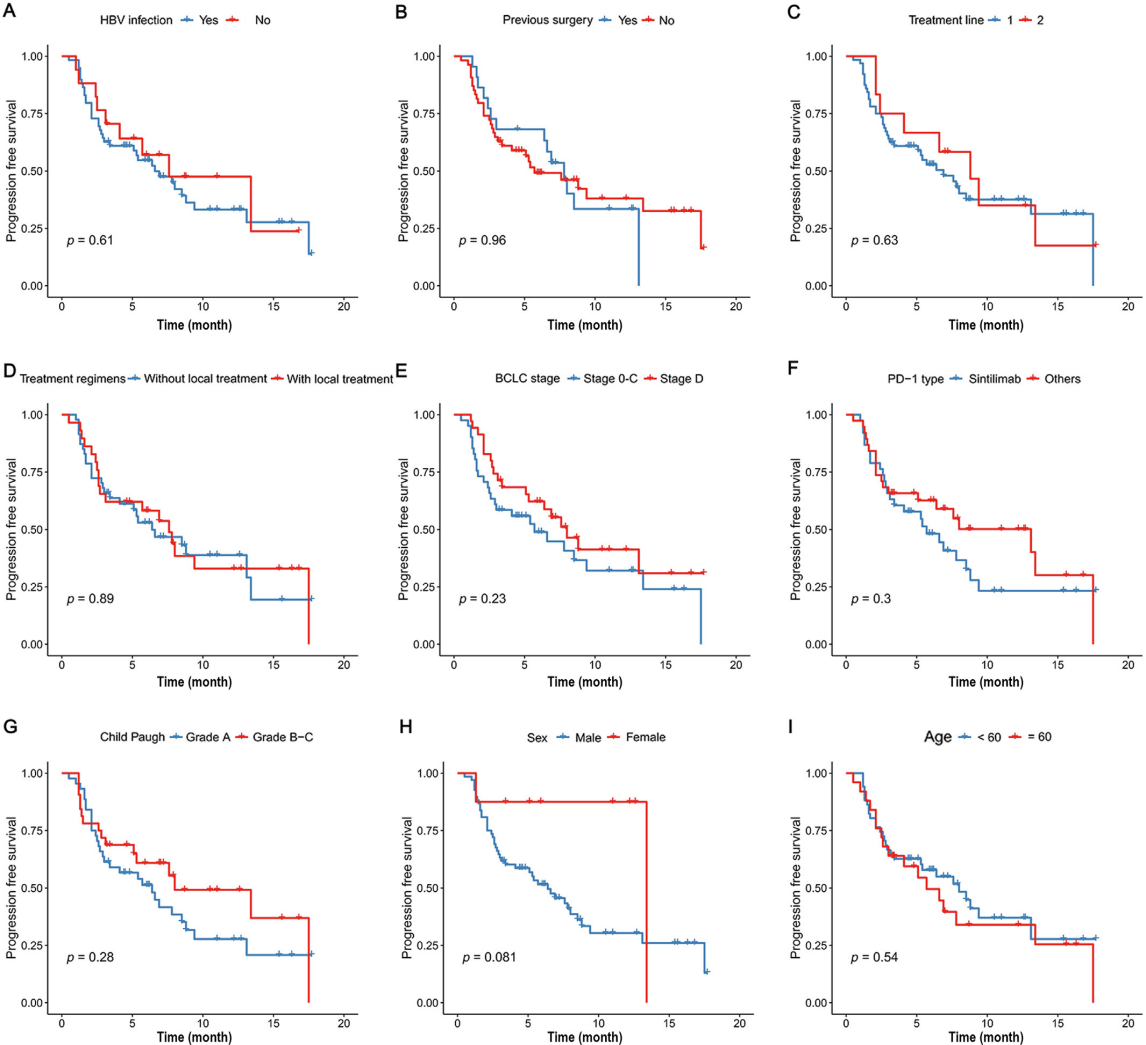
Subgroup analysis of different potential factors. (A) HBV status; (B) previous surgery; (C) treatment line; (D) treatment regimen; (E) BCLC stage; (F) PD-1 type; (G) child-paugh grade; (H) sex; (I) age.

The study conducted subgroup survival analyses based on patients' cirrhosis status, previous treatment lines, and whether they were recurrent patients. The results showed that the PFS of HCC patients without cirrhosis was longer than that of patients with cirrhosis, but no significant difference was observed. Moreover, patients who had undergone multiple previous treatment regimens did not exhibit significantly worse PFS compared with those who had received only one type of systemic treatment. Details are shown in Table 4 and Fig. 3.

**Table 4:** Univariate and multivariate analysis of baseline characteristics for overall survival and progression-free survival.

Variables	Progression-free survival (PFS)					
	Univariate Cox analysis			Multivariate Cox analysis		
	HR	95% CI	*p* value	HR	95% CI	*p* value
HBV infection (yes/no)	0.821	0.394–1.709	0.591	0.699	0.287–1.704	0.431
ECOG PS (2/0–1)	1.127	0.272–4.671	0.871	1.451	0.267–7.876	0.666
Metastasis (yes/no)	1.238	0.892–1.718	0.195	1.390	0.949–2.036	0.091
Vascular invasion (yes/no)	0.967	0.673–1.389	0.859	0.793	0.508–1.240	0.310
Recurrence after resection (yes/no)	0.967	0.519–1.875	0.987	1.041	0.529–2.046	0.908
Previous treatment (1/>2)	0.830	0.384–1.797	0.630	0.690	0.299–1.594	0.385
Combined with LT (with/without)	0.955	0.525–1.738	0.880	0.925	0.487–1.756	0.811
Cirrhosis (with/without)	0.712	0.615–1.394	0.712	0.987	0.631–1.546	0.956

Abbreviation: HR, hazard rate; CI, confidence interval; HBV: hepatitis B virus; ECOG PS: eastern cooperative oncology group performance status; LT: local treatment.

## 4 Discussion

The range of systemic treatment drugs for advanced HCC is constantly expanding, and correspondingly, the number of first-line treatment options is also increasing, leading to improved survival benefits for patients [9]. However, the overall ORR for first-line treatment remains between 4.6% and 29.8%, with 70% of patients still not achieving satisfactory treatment results [7,10]. Low response rates and frequent drug resistance are the primary reasons limiting the widespread benefits of first-line treatments. Therefore, there is an urgent need to explore second-line treatment approaches that can address the failure or progression after first-line treatments, aiming to further improve patient survival rates. Regorafenib is a tyrosine kinase inhibitor used for patients with colorectal cancer (CRC), gastrointestinal stromal tumor (GIST), and HCC. The difference between regorafenib and sorafenib is the addition of a fluorine atom on its central benzene ring, making it more pharmacologically active than sorafenib [11,12]. Xu et al. further reported that regorafenib still showed better effects than sorafenib in specific subtypes, such as cytokeratin 19-positive HCC [13]. Based on its pivotal RESORCE study, regorafenib was first approved for second-line treatment of HCC. Subsequent exploratory clinical studies have shown that sequential treatment with regorafenib after sorafenib has led to an OS of 39.4 months in patients [14,15].

PD-1/PD-L1 inhibitors could enhance the activity of immune T cells, thereby inhibiting the tumor cells. Since the prominent benefit in survival and manageable adverse effects, several PD-1/PD-L1 inhibitors have been approved for HCC treatment in clinical practice. In terms of second-line treatment, combination therapy based on PD-1 inhibitors can further improve the efficacy of single-agent anti-vascular targeting drugs [16]. Preclinical studies have shown that regorafenib combined with PD-1 inhibitors can effectively resist tumor angiogenesis, relieve immune suppression, and inhibit tumor cell proliferation and metastasis [17]. Recent studies have also demonstrated that regorafenib can optimize the efficacy of anti-PD-1/PD-L1 treatment. Regorafenib combined with nivolumab has shown favorable efficacy and tolerable safety in the treatment of CRC [18,19]. Therefore, combination therapy with regorafenib and ICIs may be a promising cancer treatment approach [20].

In addition to colorectal carcinoma, gastric cancer (GC), and biliary tract cancer, the combination of regorafenib and anti-PD-1/PD-L1 therapies has also shown promising therapeutic effects in HCC. In a multicenter retrospective study on advanced HCC, researchers evaluated the efficacy of regorafenib combined with sintilimab versus regorafenib monotherapy. Compared with single-agent therapy, combination therapy had longer PFS, higher ORR, and better OS [21,22]. The median PFS of combination therapy was 5.6 months (95% CI, 4.2–7.0 months), and the median OS was 13.4 months (95% CI, 9.2–17.5 months). The ORR of combination therapy was 24.1%, while that of monotherapy was 9.1%. In another phase Ib clinical trial of first-line treatment for advanced HCC with regorafenib combined with pembrolizumab, the DCR of the regorafenib 120 mg group was 88%, and the regorafenib 80 mg group was 91% (RECIST v1.1) [23]. In this real-world study, we set inclusion criteria about the characteristics of patients but did not intentionally restrict the type of PD-1 antibody inhibitors used. Therefore, the results of this study are relatively more generalizable. Besides, several issues about the combination therapy of PD-1 inhibitors and regorafenib were still unsolved, such as the previous systemic treatment, cirrhosis status, the types of immunotherapies, etc. This present study conducted detailed subgroup analyses to identify the most beneficial population.

This study stratified patients based on their previous systemic treatment and found no significant differences in PFS and survival between the two groups. Considering the baseline characteristics of the enrolled patients, the results potentially suggest that even though patients had previous systemic treatment, there are still benefits to receiving subsequent treatment. In terms of safety, although all patients in the study experienced adverse events, the incidence of grade 3–4 adverse events was relatively low. Compared with the findings in previous studies, no fatal adverse reactions were observed in this combination therapy for second-line treatment of HCC. Currently, available meta-analyses indicate that diarrhea, fatigue, and hand-foot skin reactions are the most common adverse events caused by regorafenib monotherapy in advanced liver cancer (PLC or HCC only?) patients in Child-Pugh A and ECOG-0 stages (cite this meta-analysis).

There were several limitations to this present study. Firstly, the retrospective design is inherently biased, especially with regard to recall bias. Additionally, we selected RECIST 1.1 as the evaluating method for tumor response rather than modified RECIST (mRECIST) or immune-modified RECIST (imRECIST). In fact, with the advancement of treatment paradigms, the criteria for defining tumor response and progression are constantly being improved and accompanied by controversies. Unusual responses associated with ICIs have led to the development of imRECIST, which aims to capture objective tumor response more accurately after ICI therapy [24]. However, in a recent study on the surrogate and modified endpoints of HCC immunotherapy, the ORR derived from RECIST was associated with better OS, and RECIST-ORR was supported as an appropriate response signal and surrogate endpoint [25]. Contrary to anticipation, the ORR determined by mRECIST and imRECIST did not demonstrate predictive power for OS benefits. For populations that have undergone complex or combination treatments, further research is needed to evaluate the interference of certain factors, such as necrosis area and pseudoprogression, on the criteria for assessment measures. As a real-world study, this research set inclusion criteria for patients but did not limit the types of acceptable PD-1 inhibitors. Therefore, the results of this study are relatively more generalizable. Several issues about the combination therapy of PD-1 inhibitors and regorafenib were still unsolved, such as the previous systemic treatment, cirrhosis status, and the types of immunotherapies. The present study conducted detailed subgroup analyses to identify the population mostly likely to benefit from regorafenib. Although this study evaluated the safety and efficacy of regorafenib combined with PD-1 inhibitor in real-world treatment, more prospective clinical trials are still needed to validate the effectiveness of this regimen [26].

## 5 Conclusions

In conclusion, this real-world clinical study demonstrates that combination therapy using regorafenib and PD-1 inhibitors can provide survival benefits for advanced HCC patients who have previously undergone at least one cycle of systemic treatment. The safety profile of this combination therapy regimen appears to be relatively manageable.

## Funding

This work was supported by the 10.13039/501100001809National Natural Science Foundation of China under contract No. 82203000, the 10.13039/501100007129Shandong Provincial Natural Science Foundation under contract No. ZR202111120102, and the Taishan Scholars Program of Shandong Province (tsqnz20221164).

## Author contributions

Z.C.L. collected the data and wrote the manuscript. J.T.Z., C.S.Z., B.Z. and X.T.S. helped to collect literature and participated in discussions. Z.C.L. and L.L. designed and verified the study. L.L. examined the language of this study. All authors read and approved the final manuscript.

## Acknowledgments

None.

## Declaration of competing interest

The authors declare no conflict of interest.

## Data available statement

The data that support the findings of this study are available on request from the corresponding author. The data are not publicly available due to privacy or ethical restrictions.

## Ethics statement

All patients were fully informed of the objectives of this study and provided formal consent before being fully considered. The protocol of this study was compliant with the principles of the Declaration of Helsinki and was also approved by the Institutional Review Board and Ethics Committee at Shandong Cancer Hospital.

## Informed consent

Not applicable.
